# Outer membrane channel protein of an oral pathogen binds human cytokine IL-8

**DOI:** 10.1080/20002297.2017.1325206

**Published:** 2017-05-30

**Authors:** Tuuli Ahlstrand

**Affiliations:** ^a^ Department of Biochemistry, University of Turku, Turku, Finland

## Abstract

Opportunistic pathogen *Aggregatibacter actinomycetemcomitans* resides in the multispecies biofilm in dento-gingival junction. *A. actinomycetemcomitans* binds and uptakes human proinflammatory cytokines, which may increase the bacterial virulence. Outer membrane  secretin channel (here OMS), a DNA binder, is involved in uptake of DNA by *A. actinomycetemcomitans*. However, OMS homologue in *Neisseria meningitidis* binds cytokines.

ELISA was used to characterize the binding of cytokines to extramembranous domain of OMS (emOMS). Binding of IL-8 to emOMS was studied with multiple methods: EuLISA, Thermofluor and Biacore. NMR and cross-linking were used to study the interaction sites. As OMS was previously described as a DNA binder, the interaction between IL-8 and DNA was studied with EMSA.

emOMS bound multiple cytokines, IL-8 being the strongest binder with K_d_ values from nM to μM. Binding of IL-8 stabilized the structure of emOMS. NMR revealed binding to five residues in IL-8 near Lys15 that was close emOMS in the cross-linking experiment. IL-8 interacted with DNA in EMSA.

OMS might form the channel that transfers IL-8 inside the bacterial cells which could be coupled to the DNA uptake. This bacterial mechanism seems both to affect the virulence of the bacterium and have potential to interfere with the host defense by binding cytokines.

